# Comparative Analysis of Prokaryotic Extracellular Vesicle Proteins and Their Targeting Signals

**DOI:** 10.3390/microorganisms11081977

**Published:** 2023-07-31

**Authors:** Ilias Stathatos, Vassiliki Lila Koumandou

**Affiliations:** Genetics Laboratory, Department of Biotechnology, Agricultural University of Athens, 11855 Athens, Greece; hliasstathatos@hotmail.gr

**Keywords:** extracellular vesicles, bacteria, archaea, signal peptide

## Abstract

Prokaryotic extracellular vesicles (EVs) are vesicles that bud from the cell membrane and are secreted by bacteria and archaea. EV cargo in Gram-negative bacteria includes mostly periplasmic and outer membrane proteins. EVs are clinically important as their cargo can include toxins associated with bacterial virulence and toxicity; additionally, they have been proposed as efficient vaccine agents and as the ancestors of the eukaryotic endomembrane system. However, the mechanistic details behind EV cargo selection and release are still poorly understood. In this study, we have performed bioinformatics analysis of published data on EV proteomes from 38 species of bacteria and 4 archaea. Focusing on clusters of orthologous genes (COGs) and using the EggNOG mapper function, we have identified cargo proteins that are commonly found in EVs across species. We discuss the putative role of these prominent proteins in EV biogenesis and function. We also analyzed the published EV proteomes for conserved signal sequences and discuss the potential role of these signal sequences for EV cargo selection.

## 1. Introduction

Extracellular vesicle (EV) production is observed in all domains of life as a conserved phenomenon in Eukarya, Archaea, and Bacteria [1,2]. Extracellular vesicles are spherical, bilayered proteolipids with an average diameter of 20–250 nm. In prokaryotes, EV production was first observed in Gram-negative bacteria over 50 years ago, owing to the electron microscope [3]. Gram-negative bacteria possess an outer membrane, which encases the EVs that they shed and, thus, their vesicles are also known as “outer-membrane vesicles” (OMVs). EV cargo in Gram-negative bacteria includes outer membrane proteins and lipids, signaling molecules, lipopolysaccharides (LPS), periplasmic and cytoplasmic proteins, DNA, RNA, and often other pathogenesis-related factors [4]. In Gram-positive bacteria, which are surrounded by a thick and rigid cell wall composed of multiple layers of peptidoglycan, the current evidence-supported hypothesis involves the action of cell-wall-degrading enzymes that weaken the peptidoglycan layer and facilitate the release of EVs [5,6]. This process also occurs in archaea where the membrane vesicles are coated with S-layer proteins [7,8].

The majority of EV studies in bacteria have been carried out on proteobacteria, the most abundant and well-studied phylum of Gram-negative bacteria. Early studies focused on model organisms and/or pathogenic species of proteobacteria [4] such as *Escherichia coli*, *Neisseria meningitidis*, *Pseudomonas aeruginosa*, *Shigella flexneri*, *Helicobacter pylori,* and *Acinetobacter baumanii.* A sum of studies regarding bacterial species that produce EVs can be found in EVpedia, an integrated and comprehensive proteome, transcriptome, and lipidome database of EVs derived from Archaea, Bacteria, and Eukarya [9].

Extracellular vesicles of Gram-negative bacteria have been suggested to play several physiological and pathological functions in bacteria–bacteria and bacteria–host interactions. Studies have focused primarily on their use as vehicles for the transmission of bacterial toxins to eukaryotic cells. Several pathogenic bacteria, such as *E. coli*, *H. pylori*, and *Shigella dysenteriae*, produce toxins that are transmitted through EVs [10]. Additionally, EVs are reported to transport proteins between cells of the same species. For example, *P. aeruginosa* uses EVs to transport a protein that offers antibiotic resistance, β-lactamase, from one cell to another [11]. Transfer of β-lactamase effectively enables the bacterial population to share the antibiotic resistance protein, thus making the gene encoding β-lactamase dispensable. Recent research has shown the ability of EVs derived from *E. coli* resistant to β-lactam to enable the survival of cells of the same species susceptible to that antibiotic [12]. Furthermore, bacterial EVs have been reported to transport their DNA cargo into eukaryotic host cells [13] and have also been proposed as a mechanism for horizontal gene transfer between bacteria based on in vitro studies [14,15].

The biogenesis of EVs in Gram-negative and Gram-positive bacteria is different due to the presence of the outer membrane and the thick peptidoglycan layer, respectively. Gram-positive bacteria are surrounded by one membrane and a thick peptidoglycan (PG) layer. Gram-negative bacteria are surrounded by an outer membrane (OM) and an inner membrane (IM), with a cross-linked PG layer bridging the two membranes. Multiple models for EV formation have been proposed for Gram-negative bacteria [4,16,17]: (a) vesicles are generated when the outer membrane expands more quickly than the underlying peptidoglycan layer, (b) accumulation of peptidoglycan fragments in the periplasm cause increased turgor pressure, thereby increasing blebbing of the OM, (c) intercalation of charged proteins in the membrane cause enhanced repulsion between LPS molecules and membrane blebbing, and (d) membrane budding occurs when the OM–PG and OM–PG–IM interactions temporarily decrease, thus allowing the dissociation of OM and PG. In both Gram-negative and Gram-positive bacteria, it is also proposed that phage-derived endolysin can trigger the formation of EVs [18]. Despite the fact that genetic and biochemical analyses have begun to shed light on aspects of EV biogenesis [2], a conserved general mechanism for biogenesis has remained elusive [19] and, indeed, the variety of vesicle types identified has increased [18].

In this study we identify the most common proteins in EV proteomes across bacterial and archaeal species and examine the presence of classic targeting signals in EV proteins to obtain new insights on EV biogenesis and EV cargo selection.

## 2. Methods

### 2.1. Species Selection and 16S Phylogenetic Tree of the Species Studied

The EV proteomes of the selected prokaryotic species were derived from the EVpedia Database (https://evpedia.info/evpedia2_xe/ accessed on 20 January 2020) as well as literature searches. We used EVpedia to collect the accession numbers of the proteins belonging to each bacterial strain, and the sequences were obtained via Uniprot using the “Retrieve/ID Mapping” tool [20]. Data for 29 Gram negative and 9 Gram-positive bacterial species were retrieved, as shown in Table 1 (for species where EV proteome data exist for multiple strains, the EV proteomes were merged to emphasize the comparison between species). Archaeal EVs have not been studied extensively, so a database has not been established concerning the EVs they produce and their protein content. Accession numbers for EV proteins were thus collected from the relevant references. However, obsolete protein records in Uniprot were frequent; either the EV proteomes were unavailable as obsolete or minimal proteins were available. Therefore, in this study, we included 4 species [21,22], as shown in Table 1.

The bacterial 16s rRNA sequences were obtained pre-aligned from the RDP database, and Clustal Omega (https://www.ebi.ac.uk/Tools/msa/clustalo/, accessed on 1 March 2020) was used to reconstruct the phylogenetic tree (Figure S1), which was visualized with FigTree v1.4.3 (https://github.com/rambaut/figtree/releases, accessed on 1 March 2020). Since archaea would be a distinct group in the tree and their number is limited, they were not included in the tree.

### 2.2. EggNOG Mapper: Assigning Proteins to COGs, Finding Common COGs between Species, and COG Functional Assignment

For the mapping of our protein dataset to Clusters of Orthologous Groups (COGs), we used the *eggNOG-mapper* tool provided by the EggNOG database [23]. EggNOG-mapper is a tool for functional annotation of large sets of sequences based on fast orthology assignments using precomputed eggNOG clusters and phylogenies. In a fasta text file, we collected all of the EV proteomes of the 38 bacterial species. The total number of proteins used was 24,762, many of which were identical, i.e., proteins with the same accession number were found between the strains. The eggNOG-Mapper tool is programed to filter any identical accession numbers; therefore, it gave results for 13,714 nonredundant proteins, each assigned to a COG. Subsequently, 4 EV proteomes from *Archaea* were collected and treated in the same way as the bacterial ones. Finally, we parsed the data (using the Vlookup function in Excel) for common COGs between the two taxonomic domains.

### 2.3. Signal Peptide Prediction Tools

To check whether the proteins that are found in EVs are led there by a signal peptide, we used the SignalP server (https://services.healthtech.dtu.dk/services/SignalP-6.0/, accessed on 20 June 2023). SignalP 6.0 predicts the presence and location of the leader peptide cleavage site in amino acid sequences based on protein language models (LMs) that use information from millions of unannotated protein sequences across all domains of life [24]. The program is able to predict standard secretory signal peptides transported by the Sec translocon and cleaved by Signal Peptidase I, lipoprotein signal peptides transported by the Sec translocon and cleaved by Signal Peptidase II, Tat signal peptides transported by the Tat translocon and cleaved by Signal Peptidase I, Tat lipoprotein signal peptides transported by the Tat translocon and cleaved by Signal Peptidase II, and pilin or pilin-like signal peptides transported by the Sec translocon and cleaved by Signal Peptidase III. Each bacterial EV proteome was analyzed in SignalP using the default settings. Regarding our 4 archaeal species, we used PRED-SIGNAL [25], a tool that is trained to identify signal peptides in archaea. In order to examine whether the signal sequences are conserved per protein cluster, we also analyzed each cluster in SignalP using the default settings. For the clusters corresponding to archaea, PRED-SIGNAL was used.

## 3. Results

### 3.1. Taxonomic Distribution of the Species Studied

The selection of the prokaryotic organisms studied was based on the availability of their EV proteomes in the EVpedia and Uniprot Databases, supplemented by data from the original publications where needed, e.g., for the datasets from archaea. Table 1 lists the 38 species of bacteria and 4 archaea used in the present study, the size of the EV proteome of each species (filtered for duplicate accession numbers), and the number of strains of each species for which EV proteome data were merged. The majority of species studied so far in the literature correspond to Firmicutes and Proteobacteria.

### 3.2. Functional Classification of COG Proteomes and Comparison between Species

**Figure 1:**
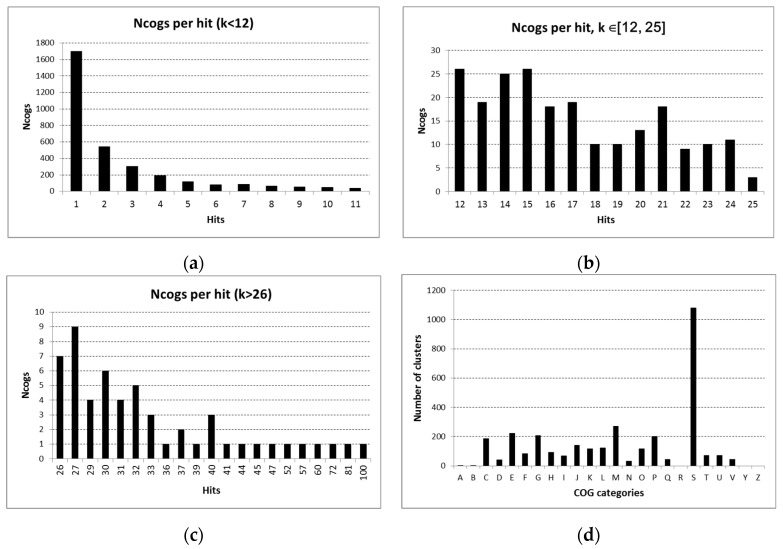
COG frequency across EV proteomes and functional classification. (**a**) Most COGs are found in only one EV proteome with decreasing number of COGs assigned to up to 12 EV proteins. (**b**) Approximately 200 COGs are each assigned to between 12 and 25 EV proteins. (**c**) A total of 55 COGs were assigned to at least 26 EV proteins; these most common COGs form the basis of the rest of the analysis (note that “hits” refer to proteins, which may be in the EV proteome of the same or different species; see Table 3 and Table 4 for the occurrence of these most common COGs across species). (**d**) Functional classification of COGs represented in all the EV proteomes analyzed (also see Table 2). The most common categories are C, E, G, M, P, and S.

In order to identify the proteins commonly found in EV proteomes across distinct species, the proteomes were analyzed using the eggNOG-Mapper tool. The results of the analysis group the orthologous proteins into clusters, with each protein assigned to one cluster. For the 13,714 total nonredundant proteins analyzed, the number of clusters formed was 3501. About half of the “clusters” contained only one protein, indicating EV proteome content which varies considerably between species (Figure 1a). Functional classification based on the categories [26] to which each cluster belongs (Table 2, Figure 1d) shows that the majority of COGs have unknown function (COG category S), while a large number of clusters belong to categories related to metabolism (categories C, E, G, and P), and, as expected, a high incidence of clusters is also seen in the “Cell wall/membrane/envelope biogenesis” category.

**Figure 2:**
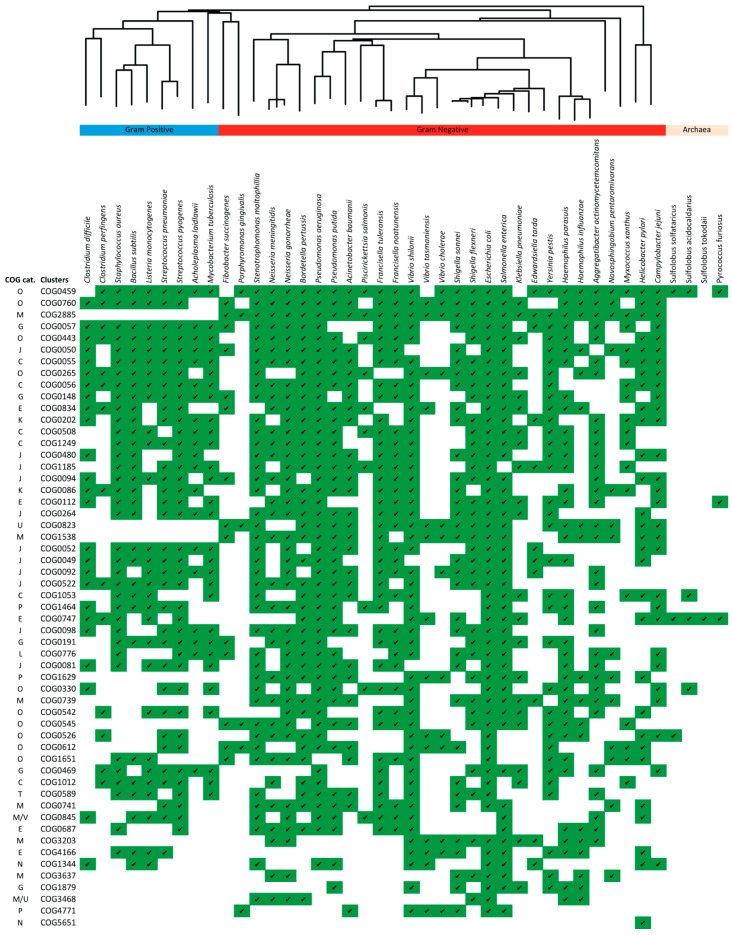
Representation of COGs in EV proteomes across species. The green-colored cells indicate that proteins for a specific COG have been identified in the corresponding species. COGs are sorted in descending order based on their occurrence in different species. Further functional information for each COG is given in Table 3. Species are ordered based on taxonomy; the 16s rRNA phylogenetic tree shown above the species names indicates their evolutionary relationships (also see Figure S1).

We focused our attention on the 55 COGs which included the highest number of proteins (minimum 26 proteins per cluster, Figure 1c), which are likely to represent proteins common to the EV proteomes of various species. The high number of proteins per COG cluster may be due to proteins being common among different species, or among different strains of one species (Figure 2). The presence or absence of corresponding clusters can be attributed, to some extent, to cell morphology (e.g., outer membrane proteins not found in Gram-positive bacteria) and to phylogenetics (Figure 2).

Table 3 gives a brief description for each of the 55 clusters, the predicted gene names coding for the corresponding proteins according to eggNOG-Mapper, and the number of species in which each cluster was identified (Identification Count). Generally, the pattern is similar to that seen in a previous study [27], mainly due to the majority of Gram-negative bacteria, but there are also proteins not identified previously (highlighted by an asterisk), such as enolase.

### 3.3. Signal Peptide Predictions

**Figure 3:**
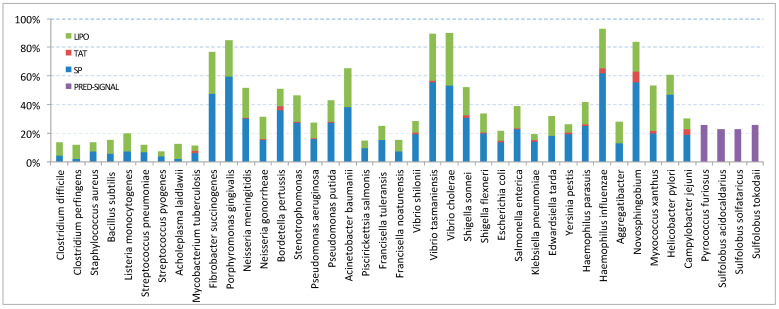
Prediction of signal sequences in the EV proteome of each species. Percentages of predicted secreted proteins per species, based on SignalP 6.0 for bacteria (SP, LIPO, and TAT) and PRED-SIGNAL for archaea. SP: standard secretory signal peptides transported by the Sec translocon and cleaved by Signal Peptidase I; LIPO: lipoprotein signal peptides transported by the Sec translocon and cleaved by Signal Peptidase II; TAT: Tat signal peptides transported by the Tat translocon and cleaved by Signal Peptidase I or Tat lipoprotein signal peptides transported by the Tat translocon and cleaved by Signal Peptidase II. For further details, including pilin or pilin-like signal peptides transported by the Sec translocon and cleaved by Signal Peptidase III, which accounted for less than 1% of the proteome in most species, see Table S2.

To check how proteins are targeted to EVs, the EV proteome of each bacterial species was tested with the SignalP 6.0 server for signal peptide prediction (Figure 3). In 8 species, at least 60% of their EV proteins have a predicted signal peptide, while, in 18 species, less than 30% of the EV proteins have a predicted signal sequence. Most predictions concern standard secretory signal peptides (SP) and lipoprotein signal peptides (LIPO). Tat signal peptides, Tat lipoprotein signal peptides, and pilin or pilin-like signal peptides accounted overall for less than 2.5% of the proteome in most species (Table S2).

Regarding the four archaeal EV proteomes, 22–26% of the proteins in each species had a signal peptide prediction using PRED-SIGNAL. The low percentage is likely due to the fact that signal prediction tools have not been studied or standardized to such an extent as they have been in bacteria. The targeting of proteins that do not have signal sequences to EVs may be mediated either by chance or attachment to other proteins (e.g., ribosomal proteins) or via nonclassical secretion.

Finally, we used SignalP and PRED-SIGNAL to investigate the signal peptides of proteins which belong to the same cluster in bacteria and archaea (Table 4). In total, 16 clusters had an overall signal sequence prediction rate of at least 60%, 7 clusters between 40 and 60%, 1 cluster (COG0845) ~15%, and 1 cluster (COG1053) ~7%, whereas 30 clusters were not predicted to include SPs.

## 4. Discussion

Systematic analysis of EV proteins may provide important insights into the mechanism of biogenesis as well as EV functions. In this study, EV proteins from 38 bacterial and 4 archaeal species were mapped to Clusters of Orthologous Groups (COGs) using eggNOG; Figure 2 and Table 3 show the 55 most frequently identified clusters, ranked in terms of how common they are among the species analyzed. Outer membrane proteins (OMPs), ribosomal proteins, transport system components, chaperones, and metabolic enzymes are the most common protein types in prokaryotic EVs.

The most common cluster (COG0459) corresponds to the chaperone GroEL (Table 3). Molecular chaperones are proteins found in abundance within the cell, which assist the covalent folding or unfolding and the assembly or disassembly of other macromolecular structures [28]. There are different types of such molecular chaperones and their sequences are highly conserved. The remaining clusters (Table 3) correspond to other chaperones (category O) and OMPs (category M, e.g., COG2885) due to the majority of the number of Gram-negative bacteria analyzed, enzymes involved in energy production/glycolysis (category C, e.g., COG0057 and COG0148), ribosomal proteins (categories J and K, e.g., COG0094 and COG0052) and enzymes involved in transcription and translation (categories J and K, e.g., COG0264 and COG0086), pathogenicity factors (categories M and N, COG0741, COG1344, and COG5651), as well as transport system components, such as the ABC transporter (categories E and G, e.g., COG0747 and COG1879).

Of interest also are conspicuous absences of these “most common” proteins from specific species or groups (Figure 2). As expected, all outer membrane bound proteins, such as OMPs (porins or receptors), Tol-Pal system components, or secretion-sensitive proteins (COG3468), are not found in Gram-positive bacteria and archaea. The most common cluster COG0459 (chaperones) is not found in *F. succinogenes, V. tasmaniensis, K. pneumoniae*, and *S. tokodaii*. COG0265 (chaperones), COG0823 (elements of the Tol-Pal system), COG1053 (oxidoreductases), COG0747 (components of the ABC system), COG0612 (Zn-dependent peptidases), and COG0589 (stress-related proteins) seem to be absent from *N. meningitides* and *N. gonorrheae*. Significant differences were also sometimes seen within the same genus; for example, in *V. shilonii*, the majority of the 55 clusters were identified, in contrast to the other two species of the *Vibrio* genus, which may be due to the origin of the strains from which the proteomes were obtained. Specifically, EV proteomes of *V. cholerae* and *V. tasmaniensis* are derived from clinical strains, while the one of *V. shilonii* comes from a strain found in coral reefs, which are complex and dynamic ecosystems capable of altering the overall load of EVs in many microorganisms [29]. Further analysis could clarify whether the absences highlight experimental differences between studies or have a functional significance for EVs in certain species.

The EV proteomes of some bacteria do not correspond well with the 55 most frequent clusters, e.g., *F. succinogenes*, *P. gingivalis*, *V. tasmaniensis*, *V. cholerae*, *E. tarda*, *N. pentaromivorans*, and *M. xanthus* (Figure 2). This may be due to non-extensive published data of the protein content of their EVs (e.g., for archaea) or due to differences or inaccuracies in the laboratory procedure for isolating EVs in the different species [17]. Despite the varied purification methods for the bacterial EVs included in this study (centrifugation, filtration, density gradient centrifugation, and tangential flow filtration), the 55 most frequent clusters are unlikely to represent impurities, as it is unlikely that the same proteins will be isolated as impurities in multiple studies.

Relatively few studies focus on how EV cargo is selected. These studies can provide mechanistic and functional insight of EV biogenesis. The majority of studies that associate the protein content of vesicles with EV biogenesis concern Gram-negative bacteria due to their pathogenicity and with the aim to use them either as drug delivery vehicles [30] or as vaccines against human pathogens such as *V. cholerae* [31], *N. meningitides* [32], etc. Apart from the outer membrane components, EV proteomes include both inner membrane proteins and a variety of cytoplasmic proteins [33], as is also seen in our analysis. Additionally, there is evidence that the VacJ/Yrb ABC transport system, a proposed phospholipid transporter, is involved in the formation of EVs [19]. This content is also verified in our study, as protein components of the ABC system were also found (clusters COG1464, COG0747, COG4166, and COG1879; Table 3).

Aiming to address the mechanism of EV cargo selection, we predicted the signal sequences of the EV cargo proteins. Until today, the general mechanism for selecting the protein content of EVs is unknown. Additionally, there are no indications that link the existence of specific signal sequences with the selection of the EV protein cargo. As shown in Figure 3, for 12 bacterial species, at least 50% of their EV proteins contain signal sequences according to SignalP. The signal prediction rate for the other species is about 25%. Particularly low rates are seen in Gram-positive bacteria, probably due to the fact that these tools have not been sufficiently trained on sequences derived from them. However, in Gram-positive bacteria, proteins may be anchored on the cell wall and, thus, exposed on the cell surface, via an LPXTG motif, targeted by membrane-associated sortase transpeptidases. It is possible that such protein can be packed into EVs and, indeed, the GPApred program [34] predicts 19 proteins with an LPXTG motif for *Clostridium difficile*, 8 of which are not found with SignalP. Still, testing GPApred on three more species (*Clostridium perfingens*, *Bacillus subtilis*, and *Acholeplasma laidlawii*) did not raise the overall prediction rate above 25% (results not shown). In general, proteins with no signal peptide could be co-purified with EVs (a) through attachment on the EV surface, e.g., via interactions with membrane-bound EV proteins, (b) packaged into EVs “by chance” after cell lysis [35], or (c) can be considered as non-classically secreted proteins (nCSPs), which are either secreted by secretion pathways which are not Sec- or Tat-dependent, such as T1SS, T3SS, T4SS [36], and T6SS [37], or by an as yet unknown mechanism [38].

The only common pattern of nCSPs is their extracellular presence (verified experimentally) despite the lack of an identifiable leader peptide or other conserved motifs [36]. Data regarding nCSPs refer to individual proteins whose secretion has been experimentally tested, but there is no indication of a wider mechanism of operation [39]. Table 5 shows the clusters for which experimental evidence exists regarding their non-classical secretion ([38] and studies within). Transport details of nCSPs beyond the boundaries of the inner membrane are not clear. Studies based on *E. coli* claim that strain properties combined with environmental and physiological stresses [38] are a possible approach to identify the secretion mechanism of nCSPs. Combining information from Table 4 and Table 5, we show in Table 6 a list of 17 clusters for which there is no information about any signal sequence or some kind of non-classical secretion. We also describe the function of each cluster (obtained from *Uniprot*) based on corresponding *E. coli* proteins belonging to each cluster. The majority of these clusters involve ribosomal proteins, which are known to lack a signal sequence. Their presence in EVs is probably due to the presence of ribosomes which are bound to the cytoplasmic membrane and interact directly with the signal recognition particle (SRP) and the Sec-YEG channel [40].

The most common EV proteins identified in this study can form a basis for testing their role in EV biogenesis, e.g., experimentally testing if their absence affects EV biogenesis. Further experiments can also discern which of these most common proteins can reliably serve as EV markers. Additionally, the information on the absence of predicted signal peptides for the majority of the EV proteins in many species opens the question of how, or indeed whether, the cargo of EVs is selected. Perhaps there is no specific overarching mechanism for the selection of EV cargo, but the nCSPs found in EVs can be analyzed bioinformatically and experimentally to look for an as yet unidentified signal which targets these proteins specifically to EVs.

## Figures and Tables

**Table 1:** Taxonomy of species included in this study. For species where EV proteome data exist for multiple strains, the EV proteomes were merged to emphasize the comparison between species. The number of proteins given for each species thus represents merged proteomes from different strains after filtering for duplicate accession numbers. * Gram-positive bacteria.

Phylum	Class	Order	Species	Proteome Size	Number of Strains
Firmicutes	Clostridia	Clostridiales	Clostridium difficile *	212	1
			Clostridium perfingens *	305	1
	Bacilli	Bacillales	Staphylococcus aureus *	563	2
			Bacillus subtilis *	235	3
			Listeria monocytogenes *	267	2
		Lactobacillales	Streptococcus pneumoniae *	638	6
			Streptococcus pyogenes *	988	1
	Mollicutes	Acholeplasmatales	Acholeplasma laidlawii *	97	1
Actinobacteria	Actinobacteria	Actinomycetales	Mycobacterium tuberculosis *	287	1
Fibrobacters	Fibrobacteria	Fibrobacterales	*Fibrobacter succinogenes*	347	1
Bacteroidetes	Bacteroidetes	Bacteroidales	*Porphyromonas gingivalis*	221	3
Proteobacteria	Beta	Neisseriales	*Neisseria meningitidis*	266	5
			*Neisseria gonorrheae*	387	5
		Burkholderiales	*Bordetella pertussis*	389	1
	Gamma	Xanthomonadales	*Stenotrophomonas maltophillia*	379	4
		Pseudomonadales	*Pseudomonas aeruginosa*	721	4
			*Pseudomonas putida*	589	1
			*Acinetobacter baumanii*	203	3
		Thiotrichales	*Piscirickettsia salmonis*	144	1
			*Francisella tuleransis*	826	2
			*Francisella noatunensis*	224	1
		Vibrionales	*Vibrio shilonii*	1366	1
			*Vibrio tasmaniensis*	132	1
			*Vibrio cholerae*	90	1
		Enterobacteriales	*Shigella sonnei*	232	1
			*Shigella flexneri*	413	2
			*Escherichia coli*	1867	6
			*Salmonella enterica*	438	2
			*Klebsiella pneumoniae*	104	2
			*Edwardsiella tarda*	72	1
			*Yersinia pestis*	270	1
		Pasteurellales	*Haemophilus parasuis*	248	1
			*Haemophilus influ* *e* *nzae*	58	2
			*Aggregatibacter actinomycetemcomitans*	151	1
	Alpha	Sphingomonadales	*Novosphingobium pentaromivorans*	129	1
	Delta	Myxococcales	*Myxococcus xanthus*	288	2
	Epsilon	Campylobacterales	*Helicobacter pylori*	299	5
			*Campylobacter jejuni*	221	2
Euryarchaeota	Thermococci	Thermococcales	*Pyrococcus furiosus*	54	1
Crenarchaeota	Thermoprotei	Sulfolobales	*Sulfolobus acidocaldarius*	31	1
			*Sulfolobus solfataricus*	44	1
			*Sulfolobus tokodaii*	27	1

**Table 2:** Functional classification of COGs represented in the EV proteomes. COG categories and descriptions derive from the COG database. The number of clusters belonging to each category is shown in the last column. Note that the numbers refer to unique protein accession numbers (after filtering out any duplicates). The most common categories are highlighted in orange (category S) and blue (categories, C, E, G, M, and P).

COG Category	Description	Number of Clusters
A	RNA processing and modification	1
B	Chromatin Structure and dynamics	1
C	Energy production and conversion	187
D	Cell cycle control and mitosis	42
E	Amino Acid metabolis and transport	222
F	Nucleotide metabolism and transport	84
G	Carbohydrate metabolism and transport	208
H	Coenzyme transport and metabolism	93
I	Lipid transport and metabolism	69
J	Translation, ribosomal structure, and biogenesis	142
K	Transcription	117
L	Replication, recombination, and repair	122
M	Cell wall/membrane/envelop biogenesis	270
N	Cell motility	32
O	Post-translational modification, protein turnover, chaperone functions	118
P	Inorganic ion transport and metabolism	200
Q	Secondary metabolites biosynthesis, transport, and catabolism	44
R	General Functional Prediction only	
S	Function Unknown	1080
T	Signal transduction mechanisms	71
U	Intracellular trafficking, secretion, and vesicular transport	70
V	Defence Mechanisms	44
Y	Nuclear structure	
Z	Cytoskeleton	

**Table 3:** Details of the most frequently occurring COGs in EV proteomes across species. COGs are sorted in descending order based on their occurrence in different species (same order as in Figure 2). Clusters which were not identified in a previous study [27] are highlighted with an asterisk.

COG	Description	Predicted Gene Names	COG Category	Identification Count
COG0459	Chaperonin GroEL (HSP60 family)	GROEL	O	37
COG0760	Parvulin-like peptidyl-prolyl isomerase	SURA, PRSA, PPID, PPID2, PPIC	O	31
COG2885	Outer membrane protein OmpA and related peptidoglycan-associated (lipo)proteins	OMPA, PAL, YFIB, YIAD, MOPB, CADF, OPRF, OCAR_4642, RMPM, MOTY	M	29
* COG0057	Glyceraldehyde-3- phosphate dehydrogenase/erythrose-4-phosphate dehydrogenase	GAP, GAPA, GAPB, EPD	G	28
* COG0443	Molecular chaperone DnaK (HSP70)	HSCA, DNAK, BMUL_5107	O	28
COG0050	Translation elongation factor EF-Tu, a GTPase	TUF	J	28
COG0055	FoF1-type ATP synthase, beta subunit	ATPD	C	28
COG0265	Periplasmic serine protease, S1-C subfamily, contain C-terminal PDZ domain	HTRA, DEGP, DEGQ, MUCD, DEGS, SCLAV_2561	O	27
COG0056	FoF1-type ATP synthase, alpha subunit	ATPA, ATPA1	C	27
* COG0148	Enolase	ENO	G	26
COG0834	ABC-type amino acid transport/signal transduction system, periplasmic component/domain	AAPJ, TCYA, SP_1394, AATB, AOTJ-1, CJAA, ARTI, FLIY, GLNH, YCKB, PATH, YXEM, PEB1, HISJ, GLNH2, GLTI, ARGT	E	25
COG0202	DNA-directed RNA polymerase, alpha subunit/40 kD subunit	RPOA	K	25
COG0508	Pyruvate/2-oxoglutarate dehydrogenase complex, dihydrolipoamide acyltransferase (E2) component	PDHC, SUCB, ACEF, ACOC, LPDA, BKDB	C	24
COG1249	Pyruvate/2-oxoglutarate dehydrogenase complex, dihydrolipoamide dehydrogenase (E3) component or related enzyme	LPDA, PDHD, LPDG, YKGC, GOR, LPD, STHA	C	24
COG0480	Translation elongation factor EF-G, a GTPase	FUSA, FUSA2	J	24
* COG1185	Polyribonucleotide nucleotidyltransferase (polynucleotide phosphorylase)	PNP	J	24
COG0094	Ribosomal protein L5	RPLE	J	24
COG0086	DNA-directed RNA polymerase, beta’ subunit/160 kD subunit	RPOC	K	24
* COG0112	Glycine/serine hydroxymethyltransferase	GLYA	E	23
* COG0264	Translation elongation factor EF-Ts	TSF	J	23
COG0823	Periplasmic component of the Tol biopolymer transport system	TOLB	U	23
* COG1538	Outer membrane protein TolC	CUSC, AGGA, TOLC, LPRN, OPRM, CYAE, CMEC, HEFA, OPRM3, IBEB, BMUL_3932, MDTQ, ARPC, NODT, ENC_28990, NATC	M	22
* COG0052	Ribosomal protein S2	RPSB	J	22
COG0049	Ribosomal protein S7	RPSG	J	22
* COG0092	Ribosomal protein S3	RPSC	J	22
* COG0522	Ribosomal protein S4 or related protein	RPSD	J	22
COG1053	Succinate dehydrogenase/fumarate reductase, flavoprotein subunit	SDHA, SDHA1,FRDA	C	21
* COG1464	ABC-type metal ion transport system, periplasmic component/surface antigen	METQ	P	21
* COG0747	ABC-type transport system, periplasmic component	DDPA, GSIB, NIKA, OPPA4, DPPA, DPPA2, ENC_24750, OPPA, APPA	E	21
COG0098	Ribosomal protein S5	RPSE	J	21
* COG0191	Fructose/tagatose bisphosphate aldolase	FBA, FBAA	G	21
* COG0776	Bacterial nucleoid DNA-binding protein	IHFB, IHFA, HUP, HUPB, HUPA, HUPN	L	20
COG0081	Ribosomal protein L1	RPLA	J	20
COG1629	Outer membrane receptor proteins, mostly Fe transport	FHUA, CIRA4, OPRC, HMUR, VPA0150, DESA, PHUR, BMUL_3200, BMUL_1933, BFRI, YNCD, CJRC, TBP1, FRPB, FETA, LBPA, HMBR, CIRA5, FOXA	P	19
* COG0330	Regulator of protease activity HflC, stomatin/prohibitin superfamily	HFLC, SCLAV_3941, HFLK, CJ0268C, QMCA	O	19
* COG0739	Murein DD-endopeptidase MepM and murein hydrolase activator NlpD, contain LysM domain	NLPD, TAGE, LASA	M	19
* COG0542	ATP-dependent Clp protease ATP-binding subunit ClpA	CLPA, CLPB, CLPC, CLPE, CLPE	O	19
* COG0545	FKBP-type peptidyl-prolyl cis-trans isomerase	FKPA, MIP, FPR2, FKLB, FKBP	O	19
* COG0526	Thiol-disulfide isomerase or thioredoxin	YNEN, DSBA, BTA, TRXA, CCMG, RESA, TLPA, DHD, TRXA_2	O	18
* COG0612	Predicted Zn-dependent peptidase	PQQL, SP_2225, YMFH, SP_2224, YHJJ, PQQE, YMXG, FCOL_05235	O	18
* COG1651	Protein-disulfide isomerase	BDBD, DSBG, DSBC, SCSC, BCFH, HP_0231	O	18
* COG0469	Pyruvate kinase	PYK, PYKA, PYKF	G	18
* COG1012	Acyl-CoA reductase or other NAD-dependent aldehyde dehydrogenase	ADHE, ALDA, YWDH, GAPN, GABD, ALDH, MMSA, BETB, CALB, YFMT, GABD4, PRR, ALDB, PCD, ALDC, GABD1, ROCA, ASTD	C	17
* COG0589	Nucleotide-binding universal stress protein, UspA family	USPA, PGUG_05476, USPE, YXIE, USPD, USPG, USPF, RV1636, BMUL_5859	T	17
* COG0741	Soluble lytic murein transglycosylase and related regulatory proteins	MLTC, MLTD, SLT, PVAA, IAGB	M	17
* COG0845	Multidrug efflux pump subunit AcrA (membrane-fusion protein)	BMUL_1520, VC1675, HEFB, CZNB, YKNX, VEXA, MACA, ACRA	M/V	16
* COG0687	Spermidine/putrescine-binding periplasmic protein	POTD, POTD2, POTF	E	15
* COG3203	Outer membrane protein (porin)	OMPF, OMPD, PHOE, OMPU, POR, OMPC, OMPN, OMPT, PORA	M	14
* COG4166	ABC-type oligopeptide transport system, periplasmic component	OPPA, MPPA, YEJA, DPPE, ALIA, ALIB, SAPA, AMIA	E	14
COG1344	Flagellin and related hook-associated protein FlgL	FLIC, FLGL, FLAA	N	13
* COG3637	Opacity protein and related surface antigens	OMPX, LOM, OPA, OCAR_7162, NSPA, ROPB2	M	9
* COG1879	ABC-type sugar transport system, periplasmic component, contains N-terminal xre family HTH domain	ARAF, YPHF, RBSB, MOCB, MGLB, TORT, RBSB11, RBSB5, RBSB2	G	9
* COG3468	Type V secretory pathway, adhesin AidA	IGA, Y0567, VAG8, PRN, BAPA, Y1454, PHG, YAPE, FLU, YDHQ, YAIT	M/U	8
* COG4771	Outer membrane receptor for ferrienterochelin and colicins	FEPA, CIRA, IRGA, CFRA, IRON	P	8
* COG5651	PPE-repeat protein	OMU116	N	1

**Table 4:** Prediction of signal sequences in the most common protein clusters across EV proteomes from different species. COGs are sorted as in Table 3 in descending order based on their occurrence in different species, but only those with an overall SP-prediction rate >10% are shown.

		Gram− Bacteria		Gram+ Bacteria		Archaea	
Cluster	Identification Count	Number of Proteins	Signal %	Number of Proteins	Signal %	Number of Proteins	Signal %
COG0760	31	47	55%	10	90%		
COG2885	29	100	82%				
COG0265	27	29	72%	11	9%		
COG0834	25	56	86%	16	88%		
COG0823	23	26	58%				
COG1538	22	60	75%				
COG1464	21	23	70%	8	88%		
COG0747	21	22	82%	5	80%	10	100%
COG1629	19	81	88%				
COG0739	19	32	56%				
COG0545	19	29	66%				
COG0526	18	36	58%	4	25%	1	0%
COG0612	18	26	58%	4	0%		
COG1651	18	27	74%	3	67%		
COG0741	17	27	63%	2	0%		
COG0845	16	21	10%	6	33%		
COG0687	15	24	83%	2	0%		
COG3203	14	44	89%				
COG4166	14	19	89%	11	91%		
COG3637	9	32	75%				
COG1879	9	27	93%				
COG3468	8	37	46%				
COG4771	8	27	85%				
COG5651	1	36	67%				

**Table 5:** Clusters for which experimental evidence exists regarding their non-classical secretion ([38] and studies within). COGs are sorted as in Table 3 in descending order based on their occurrence in different species.

COG (nCSP)	Predicted Gene Names	Identification Count
COG0459	GROEL	37
COG0057	GAP, GAPA, GAPB, EPD	28
COG0443	HSCA, DNAK, BMUL_5107	28
COG0050	TUF	28
COG0148	ENO	26
COG0508	PDHC, SUCB, ACEF, ACOC, LPDA, BKDB	24
COG1249	LPDA, PDHD, LPDG, YKGC, GOR, LPD, STHA	24
COG0480	FUSA, FUSA2	24
COG0112	GLYA	23
COG0191	FBA, FBAA	21
COG0776	IHFB, IHFA, HUP, HUPB, HUPA, HUPN	20
COG0469	PYK, PYKA, PYKF	18
COG1012	ADHE, ALDA, YWDH, GAPN, GABD, ALDH, MMSA, BETB, CALB, YFMT, GABD4, PRR, ALDB, PCD, ALDC, GABD1, ROCA, ASTD	17

**Table 6:** Clusters for which there is no information about any signal sequence or some kind of non-classical secretion. Information on the function is from Uniprot.

Clusters (No SP info)	Uniprot Description
COG0055	Produces ATP from ADP in the presence of a proton gradient across the membrane.
COG0056	Produces ATP from ADP in the presence of a proton gradient across the membrane.
COG0202	DNA-dependent RNA polymerase.
COG1185	Involved in mRNA degradation.
COG0094	It is involved in the attachment of the 5S RNA into the large ribosomal subunit.
COG0086	DNA-dependent RNA polymerase.
COG0264	Associates with the EF-Tu-GDP complex and induces the exchange of GDP to GTP.
COG0052	Required for ribosomal protein S1 to bind to the 30S subunit.
COG0049	rRNA binding proteins.
COG0092	Plays a role in mRNA unwinding by the ribosome.
COG0522	One of two assembly initiator proteins for the 30S subunit, it binds directly to 16S rRNA where it nucleates assembly of the body of the 30S subunit.
COG0098	With S4 and S12 plays an important role in translational accuracy.
COG0081	One of the primary rRNA binding proteins, it binds very close to the 3’-end of the 23S rRNA.
COG0330	HflC and HflK help govern the stability of phage lambda cII protein and thereby control the lysogenization frequency of phage lambda.
COG0542	Multi-chaperone system
COG0589	Stress response
COG1344	Flagellin is the subunit protein which polymerizes to form the filaments of bacterial flagella. Important for motility and virulence.

## Data Availability

Publicly available datasets were analyzed in this study.

## Supplementary Materials

The following supporting information can be downloaded at: https://www.mdpi.com/article/10.3390/microorganisms11081977/s1, Figure S1: 16s rRNA tree of species studied; Table S1: Functional classification of the most common 55 COGs across multiple EV proteomes; Figure S2: Functional classification of the most common COGs across multiple EV proteomes; Table S2: Details of prediction of signal sequences in the EV proteome of each species, based on SignalP 6.0 for bacteria and PRED-SIGNAL for archaea.
