# Supplementary material for: Comparative Analysis of Prokaryotic Extracellular Vesicle Proteins and Their Targeting Signals
Source: Microorganisms. 2023 Jul 31;11(8):1977. doi: 10.3390/microorganisms11081977 (PMC10458587; doi:10.3390/microorganisms11081977)
Supplement: Supplementary file 1 [file microorganisms-11-01977-s001.zip › Supplementary material/FigureS1-S2_TableS1.docx]

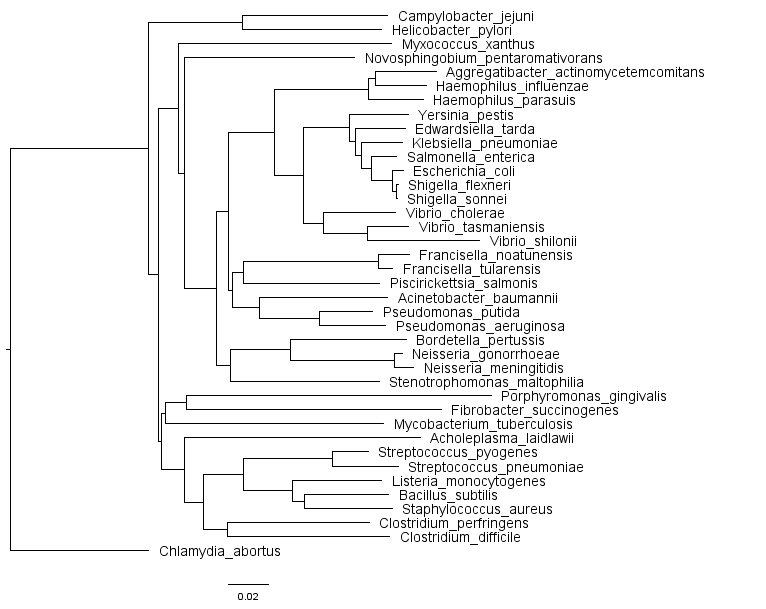


Figure S1: 16s rRNA tree of the species studied. The bacterial 16s rRNA sequences were obtained pre-aligned from the RDP database, and Clustal Omega (https://www.ebi.ac.uk/Tools/msa/clustalo/) was used to form the tree. FigTree v1.4.3 was used for imaging. Since Archaea would be a distinct group in the tree and their number is limited, they were not included in the tree.

| **COG category** | **Description** | **Number of clusters** |
| --- | --- | --- |
| A | RNA processing and modification |  |
| B | Chromatin Structure and dynamics |  |
| C | Energy production and conversion | 6 |
| D | Cell cycle control and mitosis |  |
| E | Amino Acid metabolis and transport | 5 |
| F | Nucleotide metabolism and transport |  |
| G | Carbohydrate metabolism and transport | 5 |
| H | Coenzyme transport and metabolism |  |
| I | Lipid transport and metabolism |  |
| J | Translation, ribosomal structure and biogenesis | 11 |
| K | Transcription | 2 |
| L | Replication, recombination and repair | 1 |
| M* | Cell wall/membrane/envelop biogenesis | 8 |
| N | Cell motility | 2 |
| O | Post-translational modification, protein turnover, chaperone functions | 10 |
| P | Inorganic ion transport and metabolism | 3 |
| Q | Secondary metabolites biosynthesis, transport, and catabolism |  |
| R | General Functional Prediction only |  |
| S | Function Unknown |  |
| T | Signal transduction mechanisms | 1 |
| U* | Intracellular trafficking, secretion, and vesicular transport | 1 |
| V* | Defence Mechanisms |  |
| Y | Nuclear structure |  |
| Z | Cytoskeleton |  |

Table S1: Functional classification of the most common 55 COGs across multiple EV-proteomes. COG categories and descriptions derive from the COG database. The number of clusters belonging to each category is shown in the last column. The most common categories are highlighted in blue (categories, C, E, G, J, M, O). *Two clusters which are annotated as belonging to both the M and V or U categories, respectively (see Table 3), are only counted in the M category here.

Figure S2: Functional classification of the most common COGs across multiple EV-proteomes. COG categories and descriptions derive from the COG database. The number of clusters belonging to each category is shown in Table S1. J: Translation, ribosomal structure and biogenesis; O: Post-translational modification, protein turnover, chaperone functions; M: Cell wall/membrane/envelope biogenesis; C: Energy production and conversion; E: Amino Acid metabolism and transport; G: Carbohydrate metabolism and transport; P: Inorganic ion transport and metabolism; K: Transcription; N: Cell motility; L: Replication, recombination and repair; T: Signal transduction mechanisms; U: Intracellular trafficking, secretion, and vesicular transport.
